# Identification of Novel Circulating miRNAs in Patients with Acute Ischemic Stroke

**DOI:** 10.3390/ijms23063387

**Published:** 2022-03-21

**Authors:** Eman K. Aldous, Salman M. Toor, Aijaz Parray, Yasser Al-Sarraj, Ilhame Diboun, Essam M. Abdelalim, Abdelilah Arredouani, Omar El-Agnaf, Paul J. Thornalley, Naveed Akhtar, Sajitha V. Pananchikkal, Ashfaq Shuaib, Nehad M. Alajez, Omar M. E. Albagha

**Affiliations:** 1 College of Health and Life Sciences (CHLS), Hamad Bin Khalifa University (HBKU), Qatar Foundation (QF), Doha P.O. Box 34110, Qatar; eymaldous@hbku.edu.qa (E.K.A.); mstoor@hbku.edu.qa (S.M.T.); yalsarraj@qf.org.qa (Y.A.-S.); idibou01@mail.bbk.ac.uk (I.D.); emohamed@hbku.edu.qa (E.M.A.); nalajez@hbku.edu.qa (N.M.A.); 2 Diabetes Research Center, Qatar Biomedical Research Institute (QBRI), Hamad Bin Khalifa University (HBKU), Qatar Foundation (QF), Doha P.O. Box 34110, Qatar; aarredouani@hbku.edu.qa (A.A.); pthornalley@hbku.edu.qa (P.J.T.); 3 The Neuroscience Institute, Academic Health System, Hamad Medical Corporation (HMC), Doha P.O. Box 3050, Qatar; aparray@hamad.qa (A.P.); nakhtar@hamad.qa (N.A.); spananchikkal@hamad.qa (S.V.P.); 4 Neurological Disorders Research Center, Qatar Biomedical Research Institute (QBRI), Hamad Bin Khalifa University (HBKU), Qatar Foundation (QF), Doha P.O. Box 34110, Qatar; oelagnaf@hbku.edu.qa; 5 Division of Neurology, Department of Medicine, University of Alberta, Edmonton, AB T6G 2R3, Canada; shuaib@ualberta.ca; 6 Department of Neurology, Hamad Medical Corporation (HMC), Doha P.O. Box 3050, Qatar; 7 Translational Cancer and Immunity Center, Qatar Biomedical Research Institute (QBRI), Hamad Bin Khalifa University (HBKU), Qatar Foundation (QF), Doha P.O. Box 34110, Qatar; 8 Rheumatology and Bone Disease Unit, Centre for Genomic and Experimental Medicine, Institute of Genetics and Cancer, University of Edinburgh, Edinburgh EH4 2XU, UK

**Keywords:** microRNA, miRNA, ischemic stroke, biomarkers

## Abstract

Ischemic strokes are associated with significant morbidity and mortality, but currently there are no reliable prognostic or diagnostic blood biomarkers. MicroRNAs (miRNAs) regulate various molecular pathways and may be used as biomarkers. Using RNA-Seq, we conducted comprehensive circulating miRNA profiling in patients with ischemic stroke compared with healthy controls. Samples were collected within 24 h of clinical diagnosis. Stringent analysis criteria of discovery (46 cases and 95 controls) and validation (47 cases and 96 controls) cohorts led to the identification of 10 differentially regulated miRNAs, including 5 novel miRNAs, with potential diagnostic significance. Hsa-miR-451a was the most significantly upregulated miRNA (FC; 4.8, FDR; 3.78 × 10^−85^), while downregulated miRNAs included hsa-miR-574-5p and hsa-miR-142-3p, among others. Importantly, we computed a multivariate classifier based on the identified miRNA panel to differentiate between ischemic stroke patients and healthy controls, which showed remarkably high sensitivity (0.94) and specificity (0.99). The area under the ROC curve was 0.97 and it is superior to other current available biomarkers. Moreover, in samples collected one month following stroke, we found sustained upregulation of hsa-miR-451a and downregulation of another 5 miRNAs. Lastly, we report 3 miRNAs that were significantly associated with poor clinical outcomes of stroke, as defined by the modified Rankin scores. The clinical translation of the identified miRNA panel may be explored further.

## 1. Introduction

Stroke is the second highest cause of death by disease and the leading cause of disability globally [1]. It is recognized as a neurological disorder associated with cerebrovascular anomalies, which lead to cell death in the brain via disruption in blood supply (ischemic stroke) or rupture/abnormal vasculature (hemorrhagic stroke) [2]. Importantly, cerebral injury in stroke involves neuronal cell loss and damage to astrocytes and white matter, which may cause devastating immediate and long-term effects [3]. Ischemic strokes account for around 80% of all stroke cases and comprise large-artery atherosclerosis, cardioembolic, small-vessel occlusion, and strokes of other determined or undetermined etiologies [4]. Deciphering the dynamic gene expression changes during stroke has the potential for improving disease management.

MicroRNAs (miRNAs) comprise small noncoding RNAs, which can regulate a multitude of cellular and molecular pathways and may be used as diagnostic or prognostic biomarkers for various human pathologies due to their high stability in peripheral blood [5]. Stroke diagnosis and treatment selection is predominantly dependent on clinical diagnosis and neuroimaging. However, the biochemical and molecular changes induced by stroke provide opportunities to explore and identify novel circulating blood biomarkers for diagnosing, differentiating between stroke subtypes, characterizing occlusions, and treatment selection for reperfusion therapies. While proteins such as brain natriuretic peptide (BNP), matrix metalloproteinase-9 (MMP9), and glial proteins, including GFAP and S100β, have been identified as potential biomarkers for stroke [6,7], accumulating data have explored circulating miRNAs as biomarkers for acute stroke. Notably, studies have shown variations in multiple circulating miRNAs in patients with acute ischemic stroke [8]. However, identifying specific miRNAs in clinical samples and probing their potential molecular targets may identify the pathways affected by stroke. In addition, the risk of stroke is significantly increased within 48 h of transient ischemic attack (TIA), which may not be predictable by clinicopathologic factors [9], and miRNA changes may be helpful in the prediction of patients at high risk of stroke in the early days following an acute stroke.

In the present study, we performed comprehensive circulating miRNA profiling of patients with ischemic stroke and compared their levels with healthy controls. Importantly, we also collected follow-up samples from the same patients to identify sustained dysregulation of circulating miRNAs. The most significant miRNA found was hsa-miR-451, which was upregulated in stroke patients compared with healthy controls, whereas 9 other miRNAs were significantly downregulated. Moreover, the upregulation of hsa-miR-451a and the downregulation of 5 other miRNAs was sustained in follow-up patients, indicating the persistence of their impact. Notably, our panel of dysregulated miRNAs in stroke patients showed remarkably high discriminatory performance between stroke patients and healthy controls. We also explored the potential targets of these miRNAs and found associations with previously reported stroke-related pathways. Lastly, we identified 3 miRNAs that were associated with poor clinical outcomes of stroke as assessed by the modified Rankin scale (mRS). Overall, findings of this study highlight unique circulating miRNAs in patients with ischemic stroke that could be used as diagnostic biomarkers for disease onset and may be exploited further to restore normal physiological pathways for therapeutic benefits.

## 2. Results

### 2.1. Study Design

**Figure 1:**
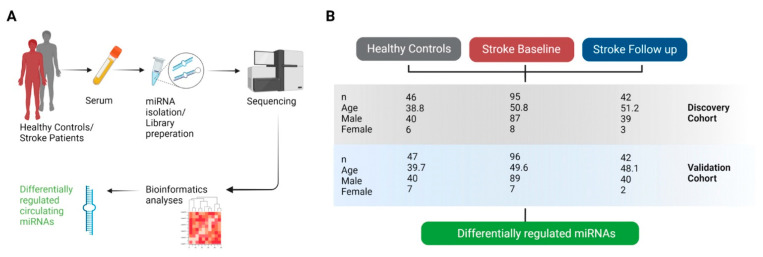
Study design. (**A**) Serum samples from stroke patients and healthy controls were collected to isolate circulating miRNAs and generate libraries for RNA-Seq. Multiple bioinformatics tools were utilized for analyses and visualization of sequencing data. (**B**) Study populations included healthy controls, stroke baseline (BL), and stroke follow-up (FU) patients. Each study population was randomly divided into discovery and validation (replication) cohorts for downstream analyses for the identification of differentially regulated miRNAs.

Serum samples from patients with stroke (base line and follow-up) and healthy controls were analyzed for circulating miRNAs for identifying differentially regulated miRNAs. An overview of the study design is depicted in Figure 1. The workflow involved random allocation of each study population into discovery and validation cohorts, with comparable distribution of covariates (age and gender). The circulating miRNA profiles of stroke patients and healthy controls were first compared between discovery cohorts considering age and gender as covariates, and the analysis then replicated in validation cohorts. The overlapping differentially regulated miRNA transcripts between discovery and validation cohorts were reported as validated miRNAs in each comparison.

### 2.2. Identification of Differentially Regulated Circulating miRNAs in Discovery and Validation Cohorts

**Figure 2:**
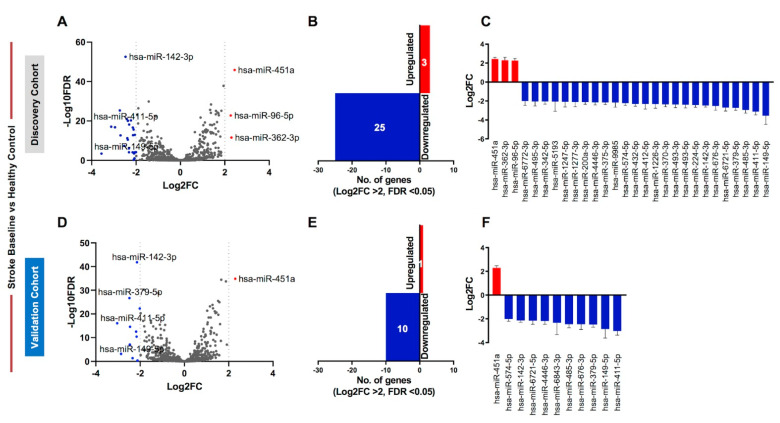
Circulating miRNA profiling of stroke baseline patients compared with healthy controls. (**A**) Volcano plot shows overall differentially regulated miRNAs in stroke baseline patients from the discovery cohort; red dots represent upregulated, blue represent downregulated, and gray dots represent unchanged miRNAs based on significance (FDR < 0.05) and log_2_FC > 2 (dotted vertical lines). (**B**) Bar plot represents the number of upregulated (red bar) and downregulated (blue bar) miRNAs using the specified cutoffs. (**C**) Column graph shows the Log_2_FC + standard error of the mean (SEM) of the 3 upregulated and 25 downregulated miRNAs in the discovery cohort. (**D**) Volcano plot, (**E**) bar plot, and (**F**) column graph show the 1 upregulated and 10 downregulated miRNAs in the validation cohort.

We compared the circulating miRNA profiles of serum samples from stroke baseline (BL) patients with healthy controls. We first compared the miRNA profiles of stroke BL patients with healthy controls in the discovery cohorts (Figure 2A–C) and found 195 differentially regulated miRNAs (fold change; FC > 2 and false discovery rate; FDR < 0.05). Using a more stringent criteria of log fold change ≥ 2 (Log_2_FC ≥ 2) revealed upregulation of 3 miRNAs and downregulation of 25 miRNAs in stroke BL patients compared with healthy controls in the discovery cohort (Figure 2A–C). Next, we repeated the analysis in the validation cohort (Figure 2D–F) and found 138 differentially expressed miRNAs (FC > 2 and FDR < 0.05), of which 11 miRNAs showed differential expression at a more stringent cutoff of Log_2_FC ≥ 2 (Figure 2D–F). 

### 2.3. Validated Differentially Regulated Circulating miRNAs in Stroke BL Patients Compared with Healthy Controls

**Figure 3:**
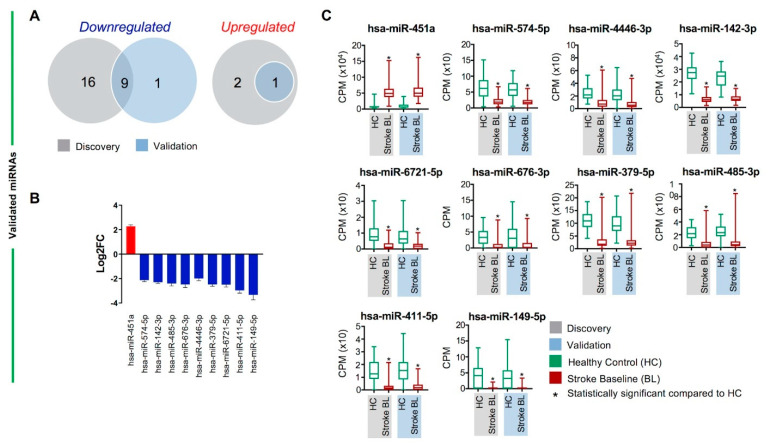
Validated differentially regulated circulating miRNAs between stroke baseline patients and healthy controls. (**A**) Venn diagram shows the total number of overlapping downregulated and upregulated miRNAs in the stroke baseline versus healthy control comparison between discovery (gray) and validation (light blue) cohorts. (**B**) Column graph shows the Log_2_FC + standard error of the mean (SEM) of 1 upregulated and 9 downregulated validated miRNAs. (**C**) Box and whiskers plots show the difference in counts per million (CPM) in stroke baseline (BL) and healthy controls (HC) in discovery (gray) and validation (light blue) cohorts of the 10 differentially regulated, validated miRNAs in stroke BL versus healthy controls. Mean with minimum and maximum values, and upper and lower quartiles are depicted for each data set with significant comparisons annotated by an asterisk (*) on top (*p* < 0.0001).

For robust identification of differentially regulated miRNAs between stroke BL patients and healthy controls, we investigated the overlapping miRNAs between discovery and validation cohorts. Combined, 123 miRNAs showed overlap between discovery and validation datasets (FC > 2 and FDR < 0.05, Supplementary Table S1). However, using a more stringent cutoff of Log_2_FC ≥ 2, results revealed upregulation of 1 and downregulation of 9 miRNAs (Figure 3A,B). hsa-miR-451a was the sole miRNA validated as upregulated in stroke BL patients compared with healthy controls, whereas the 9 downregulated miRNAs included hsa-miR-574-5p, hsa-miR-142-3p, hsa-miR-6721-5p, hsa-miR-4446-3p, hsa-miR-485-3p, hsa-miR-676-3p, hsa-miR-379-5p, hsa-miR-149-5p, and hsa-miR-411-5p. We also compared the counts per million (CPM) values of the 10 validated miRNAs to disclose the significant differences between stroke BL patients and healthy controls (Figure 3C). Reassuringly, data from discovery, validation, or combined cohorts showed consistent and comparable significance (FDR) and direction of effect (Table 1). Of note, although we adjusted for differences in age and gender between stroke patients and healthy controls by including them as covariates in our analysis model, we also performed the analysis in males only from the 2 cohorts, which resulted in the identification of the same panel of 10 differentially expressed miRNAs (Supplementary Table S2). We did not perform analysis on females only due to the small number of female patients in our study cohorts.

In addition, we used the miRDB database [10] to explore the molecular targets of the validated 10 miRNAs in stroke patients in order to highlight their potential roles in stroke. Herein, we reported the top 3 molecular targets of each miRNA with the highest prediction scores (Table 2).

### 2.4. Prediction Performance of the Identified Circulating miRNAs in Stroke Patients

**Figure 4:**
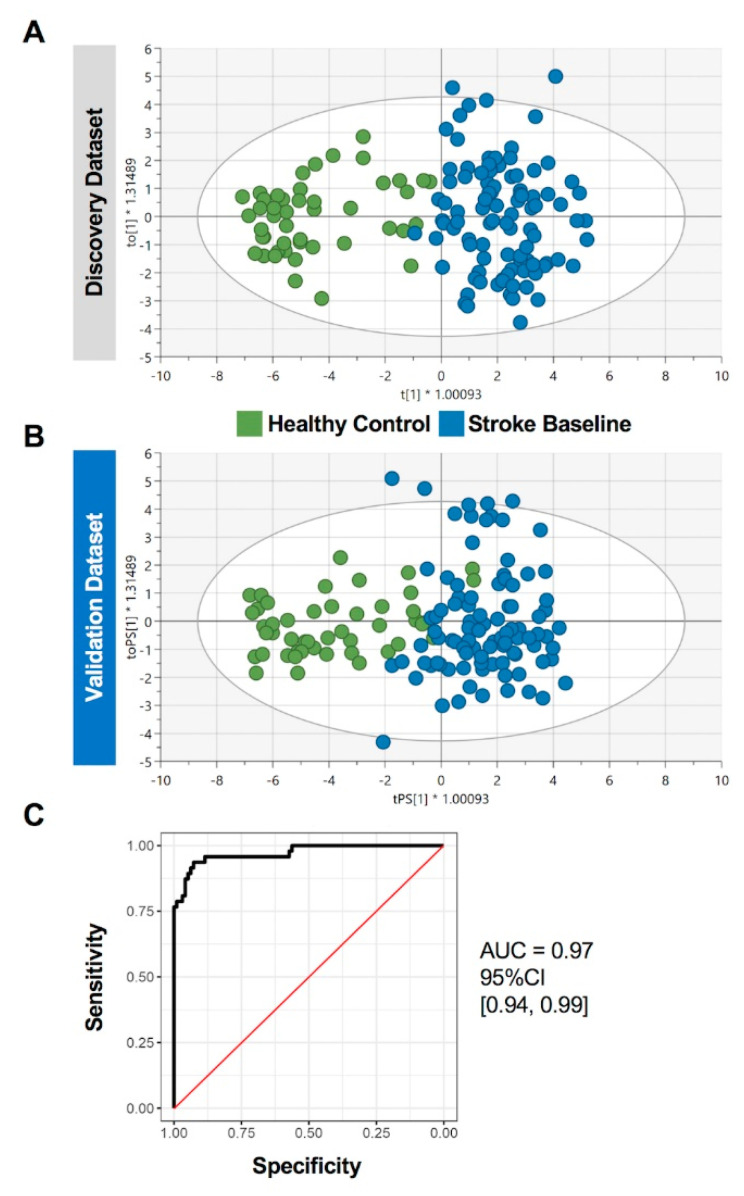
Diagnostic capacity of differentially regulated circulating miRNAs in stroke patients. The orthogonal partial-least-squares-discriminant analysis (OPLS-DA) was performed using the top differentially regulated miRNAs (n = 27) in the discovery cohort data. The classifier was trained on data from all participants in (**A**) discovery cohort (n = 142) and tested on the (**B**) validation cohort (n = 143). Scatter plots show the predictive component to discriminate stroke cases from healthy controls (green dots—*x*-axis) versus the orthogonal component representing a multivariate confounding effect that is independent of stroke (blue dots—*y*-axis). (**C**) ROC curve analysis generated an overall sensitivity of 0.94, specificity of 0.99, and AUC of 0.97.

To determine the potential diagnostic capacity of the identified dysregulated miRNA panel, we performed orthogonal partial-least-squares-discriminant analysis (OPLS-DA) in the discovery and validation cohorts (Figure 4A,B). OPLS-DA was first trained using the top differentially regulated miRNAs in the discovery cohort data (Log_2_FC ≥ 2 and FDR < 0.05, n = 27 miRNAs, Supplementary Table S3) and then tested on the validation cohort data. The classifier generated a sensitivity of 0.94, a specificity of 0.99, and an area under the curve (AUC) of 97% (Figure 4C), thereby showing high predictive ability of the identified gene panel for patients with stroke.

### 2.5. Identification of Sustained Dysregulation of Circulating miRNAs in Stroke Patients

**Figure 5:**
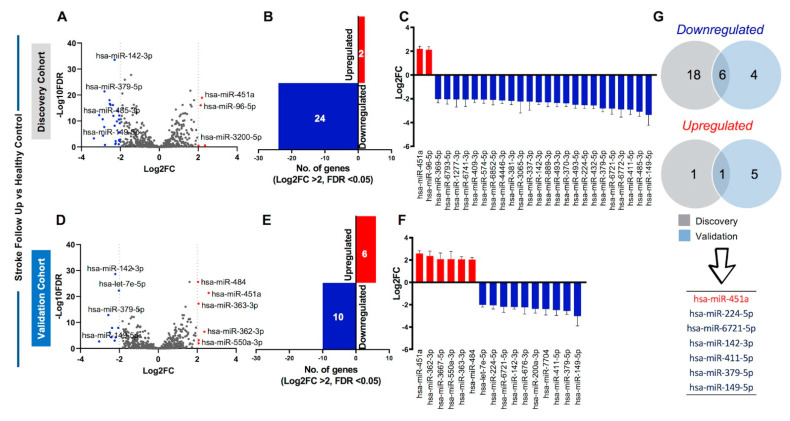
Circulating miRNA profiling of stroke follow-up patients compared with healthy controls. (**A**) Volcano plot shows overall differentially regulated miRNAs in stroke follow-up patients from the discovery cohort; red dots represent upregulated, blue represent downregulated, and gray dots represent unchanged miRNAs based on significance (FDR < 0.05) and log_2_FC > 2 (dotted vertical lines). (**B**) Bar plot represents the number of upregulated (red bar) and downregulated (blue bar) miRNA using the specified cutoffs. (**C**) Column graph shows the Log_2_FC + standard error of the mean (SEM) of the 2 upregulated and 24 downregulated miRNAs in the discovery cohort. (**D**) Volcano plot, (**E**) bar plot, and (**F**) column graph show the 6 upregulated and 10 downregulated miRNAs in the validation cohort. (**G**) Venn diagram shows the total number of overlapping 6 downregulated and 1 upregulated miRNA in the stroke follow up versus healthy control comparison between discovery (gray) and validation (light blue) cohorts. The 7 validated miRNAs are also listed.

The onset of stroke is a time-dependent event, which lasts for short periods of time but can have long-term effects on the body. Thus, to investigate the roles of dysregulated miRNAs in stroke patients over a longer period of time, we collected samples from the same clinically diagnosed stroke patients one month after diagnosis to see if there was sustained dysregulation of circulating miRNAs in stroke follow-up (FU) patients. Analysis of discovery cohort identified 145 miRNAs with FC > 2 and FDR < 0.05. However, using stringent cutoff of Log_2_FC ≥ 2, 24 miRNAs were downregulated, and 2 miRNAs were upregulated (Figure 5A–C) in the FU discovery cohort. Data from the validation cohort showed 138 miRNAs with FC > 2 and FDR < 0.05, of which 16 miRNAs (10 downregulated and 6 upregulated) showed Log_2_FC ≥ 2 (Figure 5D–F). Combined, 88 miRNAs showed overlap between discovery and validation datasets (FC > 2 and FDR < 0.05, Supplementary Table S4). Using stringent Log_2_FC criteria, 6 downregulated miRNAs and 1 upregulated miRNA was identified (Figure 5G). Moreover, data from discovery, validation, or combined cohorts in stroke FU patients also showed consistent and comparable significance (FDR) and direction of effect (Table 3). 

We then compared data from the base line and the follow-up samples and found that out of the 10 differentially regulated circulating miRNAs in stroke BL patients (listed in Table 1), 1 miRNA (hsa-miR-451a) showed sustained upregulation, while 5 miRNAs (hsa-miR-6721-5p, hsa-miR-142-3p, hsa-miR-411-5p, hsa-miR-379-5p, and hsa-miR-149-5p) showed sustained downregulation in stroke FU patients compared with healthy controls. These data indicate the prolonged involvement of these miRNAs in affecting downstream molecular targets in stroke patients.

### 2.6. Circulating miRNAs and Clinical Outcomes of Stroke

**Figure 6:**
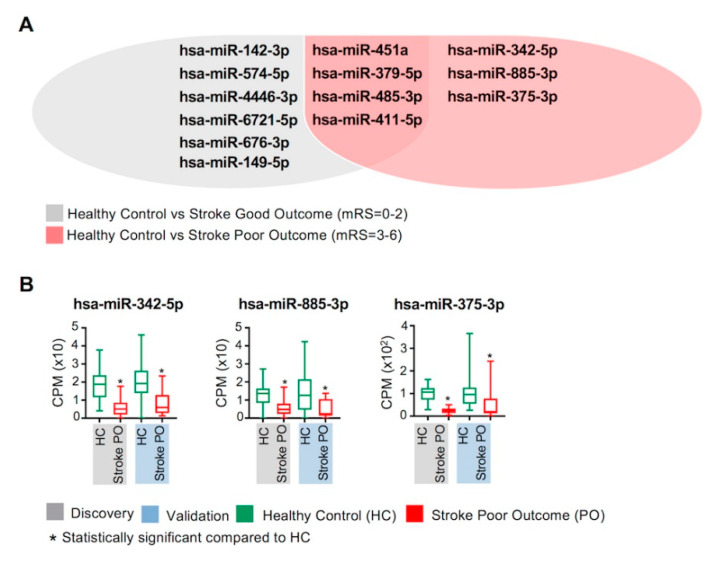
Associations between circulating miRNAs and clinical outcomes of ischemic stroke. Circulating miRNA profiles of stroke BL patients were compared with healthy controls based on the 90-day clinical follow up (mRS scores). Stroke BL patients with good outcome (discovery cohort; n = 77 and validation cohort; n = 84) and poor outcome (discovery cohort; n = 18 and validation cohort; n = 12) were compared with healthy controls (discovery cohort; n = 46 and validation cohort; n = 47). (**A**) Venn diagram lists the differentially regulated and overlapping circulating miRNAs in healthy controls versus stroke good outcome and stroke poor outcome patients. (**B**) Box and whiskers plots show the difference in counts per million (CPM) in stroke poor outcome (PO) and healthy controls (HC) in discovery (gray) and validation (light blue) cohorts of the 3 differentially regulated miRNAs, which were unique to stroke poor outcome patients. Mean with minimum and maximum values, and upper and lower quartiles are depicted for each data set with significant comparisons annotated by an asterisk (*) on top (*p* < 0.0005).

To identify circulating miRNAs that may be associated with clinical outcomes of stroke, we divided patients into two groups based on 90-day prognosis as assessed by the modified Rankin scores (mRS) (Supplementary Table S5); stroke good outcome (stroke GO; mRS = 0 to 2), and stroke poor outcome (stroke PO; mRS = 3 to 6), and compared their miRNA profiles with healthy controls (Figure 6). 

We found that the 10 differentially regulated miRNAs identified from the comparison of stroke BL patients with healthy controls (Table 1) were also validated in the analysis of stroke BL patients with good outcome versus healthy controls (Figure 6A). On the other hand, we identified 7 differentially regulated miRNAs between poor outcome stroke patients and healthy controls. Importantly, out of these 7 miRNAs, 3 (hsa-miR-342-5p, hsa-miR-885-3p and hsa-miR-375-3p) were exclusively downregulated in stroke patients with worse prognosis (Figure 6A,B). These data reflect the association between these 3 miRNAs and poor clinical outcomes of ischemic stroke.

## 3. Discussion

In this study, we identified 10 differentially regulated circulating miRNAs in stroke patients compared with healthy controls, of which miR-451a, miR-574-5p, miR-142-3p, miR-411-5p, and miR-379-5p were previously associated with stroke, while the other 5 (miR-676-3p, miR-149-5p, miR-4446-3p, miR-6721-5p, and miR-485-3p) are novel. Notably, hsa-miR-451a was the most significantly upregulated miRNA in stroke patients (Log_2_FC: 2.27). In agreement with our findings, miR-451a has been previously shown to be elevated in patients with acute ischemic stroke and transient ischemic attack (TIA) patients [11]. In addition, Kong et al. showed that miR-451a is upregulated in circulating natural killer (NK) cells in ischemic stroke patients and its inhibition enhances NK cell activation [12]. To elucidate the role of miR-451a in stroke, we explored its molecular targets. Interestingly, we found that the top 3 molecular targets of miR-451a (*OSR1*, *CUX2*, *PSMB8*) have been previously associated with stroke or studied in relation with brain damage. WNK3-SPAK/OSR1 cation-chloride cotransporter pathway has been reported as a potential therapeutic target in ischemic stroke as knocking down SPAK/OSR1 improved neuroprotection [13]. *CUX2* has been reported to be associated with atrial fibrillation in Japanese populations [14] and also as a risk factor for ischemic stroke [15], while PSMB8 is a component of immunoproteasome LMP7, which is elevated in ischemic stroke and contributes to neuroinflammation [16]. Combined, these data show the dynamic roles of miR-451a in ischemic stroke and support its utilization as a biomarker for disease identification. Moreover, the sustained upregulation of miR-451a in stroke FU patients demonstrates its ongoing and active modulation of stroke-affected pathways. 

Among the downregulated miRNAs in our study, miR-574-5p, miR-142-3p, miR-411-5p, and miR-379-5p have previously been linked to stroke and cerebral injury. In line with our results, hsa-miR-574-5p was found to be downregulated in stroke patients and has been proposed as a biomarker for stroke diagnosis [17], while single nucleotide polymorphisms (SNPs) in one of its targets, *FOXI2*, has been identified as a risk factor for large vessel ischemic stroke [18]. Hsa-miR-142-3p was significantly downregulated in various stroke subtypes [19], consistent with our data, and expression of its target *ZEB2* has been reported to be significantly increased following ischemic stroke [20]. Moreover, hsa-miR-411-5p was slightly upregulated in acute ischemic stroke patients receiving recombinant tissue plasminogen activator therapy compared with untreated patients [21]. However, our data shows downregulation of miR-411-5p and differences in study design could account for these discrepancies. Lastly, in accordance with our findings, miR-379-5p was downregulated in patients with ischemic stroke [22], while its neuroprotective roles in targeting MAP3K2 and JNK/c-Jun signaling to attenuate neuronal autophagy were also reported recently [22].

Our analysis identified five novel downregulated miRNAs in stroke patients (miR-676-3p, miR-149-5p, miR-4446-3p, miR-6721-5p, and miR-485-3p). Although these were not previously reported to be dysregulated in stroke, some of their molecular targets have been associated with stroke pathology. For example, the molecular target of hsa-miR-676-3p, *SMURF2*, is involved in neurodifferentiation in recovery phase following ischemic stroke [23], while its other targets, *PTPRB* [24] and *ANP32B* [25], have also been previously investigated in experimental stroke models. Notably, *PTPRB* encodes the protein tyrosine phosphatase receptor type B (also known as vascular endothelial protein tyrosine phosphatase—VE-PTP), which is involved in the maintenance of vascular integrity and is a potential therapeutic target for vascular diseases, including stroke [26]. Targeting VE-PTP triggers blood vessel enlargement and averts vascular leakage via Tie-2 signaling, which may aid drug delivery across the blood–brain barrier and also prevent cerebral leakage and edema [27]. Additionally, the neuroprotective role of *KIF2A*, the molecular target of hsa-miR-149-5p, has been attributed to NF-kB pathway and presented as a therapeutic target for cerebral ischemic injury [28]. Moreover, *CBX7* [29] (target of miR-4446), *SPRN* [30] and *STK35* [31] (targets of miR-6721-5p) have also been studied in stroke models. *SPRN* encodes the shadow of prion protein, associated with neurodegenerative human prion diseases including Creutzfeldt–Jakob disease [32], while knockdown of *STK35* in endothelial cells alleviates their migratory ability [33]. Thus, downregulation of these miRNAs in patients with ischemic stroke may affect important pathways associated with neuroprotective roles and preservation of adequate vasculature. In addition, while miR-485-3p was significantly dysregulated in ischemic rat brain [34] and has been potentially associated with the severity of heart failure [35], its levels and its molecular targets have not been explored in relation to stroke. 

Furthermore, some of the miRNAs reported herein have been previously associated with other pathological conditions. For instance, hsa-miR-451a has been reported as a tumor suppressor in gastric cancer [36] and hepatocellular carcinoma [37], hsa-miR-574-5p has been proposed as a biomarker for non-small cell lung cancer [38] and as a metabolic regulator in gestational diabetes mellitus [39], while dysregulation of hsa-miR-6721-5p, hsa-miR-142-3p, and hsa-miR-149-5p has been reported in Alzheimer’s disease [40].

Previous studies have predominantly applied quantitative real-time PCR or microarray analyses on serum samples from stroke patients to identify potential biomarkers. For instance, investigating circulating miRNA levels in ischemic stroke patients compared with healthy controls showed downregulation and selective upregulation of certain miRNAs in other types of ischemic stroke [41]. In addition, selective exosomal miRNAs were shown to be significantly increased in ischemic stroke, with some miRNAs showing potentials of differentiating between early acute phase and recovery phase [42,43], while elevated serum expression levels of certain miRNAs showed correlation with high sensitivity C-reactive protein (hs-CRP) and MMP-9 in ischemic stroke patients [44]. In addition, some miRNAs have been reported to potentially differentiate between ischemic stroke and TIA patients [45]. In contrast, comprehensive miRNA profiling via RNA-Seq to detect a wider range of miRNA transcripts in stroke patients remains largely unexplored. For instance, Tiedt et al., performed RNA-Seq on a modest sample size (n = 20) of patients with ischemic stroke compared with healthy controls (n = 20) and validated their findings in a larger cohort (n = 200) by RT-PCR and reported 3 miRNAs as potential biomarkers for acute ischemic stroke with sensitivity and specificity superior to routine imaging techniques [46]. He et al. investigated the prognostic significance of miRNAs in patients with acute ischemic stroke receiving reperfusion therapies and exhibiting varying disease outcomes (n = 10), and reported associations between elevated levels of selective miRNA levels and adverse outcomes [47]. Recently, Mens et al. analyzed samples from the Rotterdam study [48], and identified 3 miRNAs that were associated with increased risk of stroke [49]. However, these miRNAs are not observed in our list of differentially expressed miRNAs likely due to differences in study design as many of the previously reported studies were retrospective. In this study, we used strict analysis criteria (cutoffs) and designed our study (sample collection timepoints and workflow) to ensure identification of miRNAs with robust diagnostic significance.

While miRNAs are increasingly being explored as drug targets for cardiovascular conditions [50], accumulating evidences in pharmacogenomics have shown their potential involvement in drug response [51]. Patients in our study cohort were prescribed anti-coagulants (~98%), anti-platelet drugs (~90%), statins (~96%), anti-hypertensive drugs (~69%), and anti-diabetic medication (~44%) for disease management. Although studies have shown that these classes of drugs can potentially affect selected miRNAs [52,53,54,55], the sustained dysregulation of 6 out of 10 differentially expressed miRNAs in stroke follow-up patients provided strong evidence that these miRNAs were not affected by these drugs. However, the remaining 4 miRNAs could be potentially affected by these drugs, but these observations require further investigation and validation, and may be considered as a limitation of our study.

The modified Rankin scale (mRS) is utilized as a common clinical tool for assessing outcomes for stroke [56] due to its high reliability in evaluating disease outcome [57]. mRS scores ranging from 0 to 2 indicate good outcomes based on daily activity performance [58]. We found that stroke patients with good outcome showed dysregulation in the same 10 miRNAs as our initial comparison of stroke BL patients with healthy controls. In contrast, while stroke patients with poor outcome also showed dysregulation of 4 miRNAs out of the initial 10 dysregulated miRNAs, dysregulation of 3 miRNAs (miR-342-5p, miR-885-3p, and miR-375-3p) was only observed in stroke patients with poor outcomes. The downregulation of hsa-miR-342-5p has been previously reported in ischemic stroke [59] and in the recovery phase of stroke [17]. Moreover, hsa-miR-375-3p has been shown to be downregulated in ischemic reperfusion injury models and involved in neuroprotective roles [60]. In contrast, although hsa-miR-885-3p has been shown to be upregulated in a mouse model of cerebral ischemia treated with valproic acid [61] and increased in induced status epilepticus [62], it has not been previously reported in ischemic stroke patients. Overall, our findings reflect the potential roles of these miRNAs in the clinical manifestation/poor outcomes of patients with ischemic stroke. 

The discriminative capacity of the miRNA identified in this study (AUC = 0.97) was superior to previously reported biomarkers of acute ischemic stroke such as C-reactive protein (AUC 0.73, [63,64]), interleukin-6 (AUC = 0.82, [65]), and neuron-specific enolase: (AUC = 0.69, [66]). High accuracy to discriminate stroke from healthy controls based on miRNA profiling shows promising clinical application. Our study design involved comprehensive investigation of circulating miRNA profiles from clinical samples from a larger cohort and analysis workflow ensured robust identification of differentially expressed miRNAs in stroke patients compared with healthy controls. To ensure high validity and efficiency of our findings, our study design involved randomization of samples and division into discovery and validation cohorts and using stringent cutoffs in our analysis. However, validation in a larger external dataset is warranted to strengthen our findings. Importantly, functional studies to examine the biological significance of the identified novel miRNA panel in the pathogenesis of cardiovascular diseases and to explore the molecular pathways affected in stroke are required. Moreover, additional data on patients’ clinical parameters and follow up clinical data would have assisted investigating additional confounder effects and in investigating correlations with other disease outcomes. Similarly, data of brain imaging would also assist in investigating differences and correlations with other cerebrovascular disorders. Overall, the differentially regulated miRNAs and their molecular targets presented herein may be explored further to elucidate their influence on stroke onset and progression.

## 4. Materials and Methods

### 4.1. Samples

Study cohorts comprised healthy controls, and clinically diagnosed ischemic stroke patients admitted to Hamad General Hospital (Doha, Qatar). Serum samples (200 µL) were collected within 24 hr (stroke baseline; BL, n = 198), 1 month after disease diagnosis (stroke follow up; FU, n = 84) and healthy controls (n = 94). Hemolyzed samples (stroke BL; n = 7, healthy controls; n = 1) were removed from the analyses. In addition, 90-day clinical follow up data for disease outcome via assessment of mRS was retrieved to classify patients with good and poor outcomes, and details for medication prescribed to stroke patients were also retrieved. 

This study was executed under ethical approvals from Qatar Biomedical Research Institute (2019-013) and Hamad Medical Corporation (15304/15), and in accordance with applicable guidelines and regulations. All participants provided written informed consent prior to sample donation. Key characteristic features of study populations are presented in Figure 1B.

### 4.2. Circulating miRNA Purification, Library Preparation, and Sequencing

Total RNA was purified from serum samples using miRNeasy Serum/Plasma Advanced Kit (Qiagen, Hilden, Germany) as per the manufacturer’s protocol. RNA concentrations were determined using Qubit RNA Broad Range Assay Kits (Invitrogen, CA, USA). Libraries were generated using QIAseq miRNA NGS Library Kit (Qiagen) according to manufacturer’s protocol. QIAseq miRNA NGS 96 Index IL kit (Qiagen) was used for indexing and the resulting libraries were quantified using Qubit dsDNA HS assay kit (Invitrogen) and its size distribution was determined using the Agilent 2100 Bioanalyzer DNA1000 chip (Agilent Technologies, Santa Clara, CA, USA). Quality-passed libraries were pooled, clustered using TruSeq PE Cluster Kit v3-cBot-HS (Illumina, San Diego, CA, USA) and sequenced using illumina HiSeq 4000 instrument at 10 million reads per sample utilizing HiSeq 3000/4000 SBS kit (Illumina) as per the manufacturer’s protocol.

### 4.3. RNA-Seq Data Processing and Analyses

Various bioinformatics tools were utilized for analyses and visualization of RNA-Seq data. Single reads (at 75 cycles) were mapped to the human miRbase v22 reference genome in CLC Genomics Workbench 21.0.5 (Qiagen). The levels of expression of transcripts were reported as the score of counts per million (CPM) of total count mapped mature miRNA reads. Data were calibrated for RNA spike-in (RNA transcript of known sequence and quantity) reads. Differential miRNA analyses were performed on RStudio (version 4.1.1; RStudio, Boston, MA, USA) using the DSeq2 method [67], using age and gender as covariates. Stringent criteria were applied to identify differentially regulated miRNAs in all comparisons (FC > 2 or Log_2_FC > 2 and false discovery rate (FDR) *p* value < 0.05). Statistical analyses and visualization of RNA-Seq data were performed using GraphPad Prism 9.1.2 (GraphPad Software, San Diego, CA, USA). miRNA targets were identified from the miRDB database [10]. 

### 4.4. Discriminant Analyses

To determine the capacity of predicted variables (differentially regulated miRNAs) to discriminate between stroke patients and healthy controls, discriminant analyses were performed using orthogonal projection to latent structure discriminant analysis (OPLS-DA) classifier using SIMCA software (version 15; Umetrics, Umeå, Sweden) on the discovery dataset from the stroke baseline versus healthy control analysis. The model was then tested on the validation dataset and the performance was assessed by generating receiver operating characteristic (ROC) curve and determining the area under the curve (AUC) value. The sensitivity and specificity constants of the test were determined based on similar classification threshold as the median of the predicted scores by the OPLS-DA classifier. 

## Figures and Tables

**Table 1:** Circulating miRNAs in stroke baseline patients versus healthy controls.

	Discovery		Validation		Combined	
miRNA	Log_2_FC ^1^	FDR ^2^	Log_2_FC ^1^	FDR ^2^	Log_2_FC ^1^	FDR ^2^
hsa-miR-451a	2.4	1.28 × 10^−46^	2.3	1.36 × 10^−35^	2.3	3.78 × 10^−85^
hsa-miR-574-5p	−2.2	5.17 × 10^−21^	−2.0	5.15 × 10^−23^	−2.1	7.25 × 10^−53^
hsa-miR-142-3p	−2.5	2.69 × 10^−53^	−2.1	1.54 × 10^−42^	−2.3	2.34 × 10^−110^
hsa-miR-6721-5p	−2.7	2.08 × 10^−13^	−2.2	4.17 × 10^−11^	−2.5	9.05 × 10^−34^
hsa-miR-4446-3p	−2.1	1.67 × 10^−13^	−2.2	3.09 × 10^−13^	−2.0	1.13 × 10^−26^
hsa-miR-485-3p	−2.9	1.71 × 10^−17^	−2.5	2.82 × 10^−15^	−2.4	1.33 × 10^−30^
hsa-miR-676-3p	−2.5	4.34 × 10^−08^	−2.5	8.88 × 10^−08^	−2.5	3.76 × 10^−21^
hsa-miR-379-5p	−2.7	4.76 × 10^−26^	−2.5	2.01 × 10^−27^	−2.5	1.67 × 10^−50^
hsa-miR-149-5p	−3.6	3.57 × 10^−04^	−2.9	7.29 × 10^−04^	−3.3	4.24 × 10^−16^
hsa-miR-411-5p	−3.1	8.11 × 10^−18^	−3.0	8.78 × 10^−17^	−3.0	1.11 × 10^−40^

^1^ Log_2_ fold change; ^2^ false discovery rate.

**Table 2:** Target genes of 10 differentially regulated circulating miRNAs in stroke baseline patients.

miRNA	Target-1	Score	Target-2	Score	Target-3	Score
hsa-miR-451a	*OSR1*	92	*CUX2*	90	*PSMB8*	90
hsa-miR-574-5p	*CALCOCO1*	100	*FOXI2*	100	*C11ORF96*	97
hsa-miR-142-3p	*ZEB2*	100	*TASOR2*	100	*RICTOR*	99
hsa-miR-6721-5p	*NECTIN1*	100	*KIF21B*	100	*SPRN*	100
hsa-miR-4446-3p	*DR1*	98	*CBX7*	96	*MBNL2*	96
hsa-miR-485-3p	*CREBRF*	100	*ELAVL2*	99	*PDZD2*	99
hsa-miR-676-3p	*PTPRB*	98	*SMURF2*	97	*ANP32B*	95
hsa-miR-379-5p	*TXLNG*	98	*MTMR2*	97	*EIF4G2*	94
hsa-miR-149-5p	*CACHD1*	98	*ELP5*	98	*VPS53*	98
hsa-miR-411-5p	*MITD1*	97	*ELFN1*	97	*RNF149*	97

**Table 3:** Circulating miRNAs in stroke follow-up patients versus healthy controls.

	Discovery		Validation		Combined	
miRNA	Log_2_FC ^1^	FDR ^2^	Log_2_FC ^1^	FDR ^2^	Log_2_FC ^1^	FDR ^2^
hsa-miR-451a	2.2	1.09 × 10^−19^	2.6	4.46 × 10^−22^	2.3	2.03 × 10^−45^
hsa-miR-224-5p	−2.5	2.68 × 10^−17^	−2.1	1.17 × 10^−08^	−2.3	2.04 × 10^−27^
hsa-miR-6721-5p	−2.8	2.07 × 10^−08^	−2.2	5.38 × 10^−05^	−2.4	1.98 × 10^−15^
hsa-miR-142-3p	−2.3	2.85 × 10^−34^	−2.2	2.16 × 10^−29^	−2.3	6.91 × 10^−77^
hsa-miR-411-5p	−2.9	2.13 × 10^−11^	−2.5	7.66 × 10^−07^	−2.8	4.52 × 10^−25^
hsa-miR-379-5p	−2.8	4.15 × 10^−22^	−2.6	1.43 × 10^−13^	−2.7	1.17 × 10^−44^
hsa-miR-149-5p	−3.4	5.20 × 10^−04^	−3.0	2.00 × 10^−03^	−3.3	1.06 × 10^−11^

^1^ Log_2_ fold change; ^2^ false discovery rate.

## Data Availability

All data generated or analyzed during this study are included in this published article (and its supplementary information files) or are available from the corresponding author by reasonable request.

## Supplementary Materials

The following supporting information can be downloaded at: https://www.mdpi.com/article/10.3390/ijms23063387/s1.
