# Supplementary material for: Identification of Novel Circulating miRNAs in Patients with Acute Ischemic Stroke
Source: Int J Mol Sci. 2022 Mar 21;23(6):3387. doi: 10.3390/ijms23063387 (PMC8955546; doi:10.3390/ijms23063387)
Supplement: Supplementary file 1 [file ijms-23-03387-s001.zip › ijms-1640996-supplementary/Supplementary_Table_S2_Stroke_BL_Males_vs_Healthy_Control_Males.pdf]

Supplementary Table S2. Circulating miRNA in stroke baseline patients (Males) versus healthy controls (Males).

	Discovery		Validation		Combined	
**miRNA**	**Log2FC[1]**	**FDR[2]**	**Log2FC[1]**	**FDR[2]**	**Log2FC[1]**	**FDR[2]**
hsa-miR-451a	2.3	2.73×10^{-31}	2.3	1.17×10^{-28}	2.2	2.59×10^{-64}
hsa-miR-574-5p	-2.2	1.97×10^{-15}	-2.2	4.85×10^{-21}	-2.1	5.12×10^{-43}
hsa-miR-142-3p	-2.4	1.04×10^{-38}	-2.1	1.68×10^{-31}	-2.3	1.03×10^{-83}
hsa-miR-6721-5p	-2.6	6.21×10^{-10}	-2.1	4.23×10^{-08}	-2.4	4.51×10^{-24}
hsa-miR-4446-3p	-2.1	4.92×10^{-10}	-2.1	2.79×10^{-09}	-1.9	2.58×10^{-19}
hsa-miR-485-3p	-2.8	7.09×10^{-13}	-2.6	3.90×10^{-14}	-2.4	1.07×10^{-24}
hsa-miR-676-3p	-2.4	1.75×10^{-05}	-2.5	2.08×10^{-06}	-2.5	9.25×10^{-16}
hsa-miR-379-5p	-2.8	1.45×10^{-19}	-2.3	2.34×10^{-19}	-2.4	3.62×10^{-39}
hsa-miR-149-5p	-3.6	8.49×10^{-03}	-3.1	1.23×10^{-02}	-3.4	2.85×10^{-10}
hsa-miR-411-5p	-2.9	9.13×10^{-13}	-3.0	1.76×10^{-13}	-2.8	3.67×10^{-31}

[1]Log2 Fold Change; [2]False Discovery Rate
