# Supplementary material for: Identification of Novel Circulating miRNAs in Patients with Acute Ischemic Stroke
Source: Int J Mol Sci. 2022 Mar 21;23(6):3387. doi: 10.3390/ijms23063387 (PMC8955546; doi:10.3390/ijms23063387)
Supplement: Supplementary file 1 [file ijms-23-03387-s001.zip › ijms-1640996-supplementary/Supplementary_Table_S5_Stroke_mRS.pdf]

Supplementary Table S5. 90-day clinical follow up data (modified Rankin Scale – mRS) of stroke baseline patients.

mRS score	Outcome	Discovery		Validation	
		n	(%)	n	(%)
0	Good	54	56.8	64	66.7
1		13	13.7	13	13.5
2		10	10.5	7	7.3
3	Poor	15	15.8	11	11.5
4		2	2.1	1	1.0
5		1	1.1	0	0.0
6		0	0.0	0	0.0
